# Review of Current Management of Myocardial Infarction

**DOI:** 10.3390/jcm14176241

**Published:** 2025-09-04

**Authors:** Trisha Saha, Hatem Soliman-Aboumarie

**Affiliations:** 1 Bloomsbury Campus, University College London Medical School, 74 Huntley St, London WC1E 6DE, UK; trisha.saha.20@ucl.ac.uk; 2 Department of Anaesthetics and Cardiothoracic Critical Care, Harefield Hospital, Hill End Road, Uxbridge, London UB9 6JH, UK; 3 School of Cardiovascular and Metabolic Medicine and Sciences, King’s College London, Denmark Hill, London SE5 9RS, UK

**Keywords:** myocardial infarction, management, cardiovascular disease, STEMI, NSTEMI, ACS

## Abstract

Myocardial infarction (MI), commonly known as a heart attack, remains a leading cause of morbidity and mortality worldwide. It arises from a sudden cessation of coronary blood flow, most often due to plaque rupture and subsequent thrombus formation. Over the years, significant advances in the understanding, diagnosis, and treatment of MI have transformed patient outcomes. This review explores the current approaches to the management of myocardial infarction, highlighting evidence-based practices, recent advancements, and ongoing challenges in optimising care across various healthcare settings.

## 1. Introduction

Myocardial infarction (MI), commonly known as a heart attack, remains a leading cause of morbidity and mortality worldwide [1]. It arises from a sudden cessation of coronary blood flow, most often due to plaque rupture and subsequent thrombus formation. Over the years, significant advances in the understanding, diagnosis, and treatment of MI have transformed patient outcomes. This review explores the current approaches to the management of myocardial infarction, highlighting evidence-based practices, recent advancements, and ongoing challenges in optimising care across various healthcare settings.

**Figure 1:**
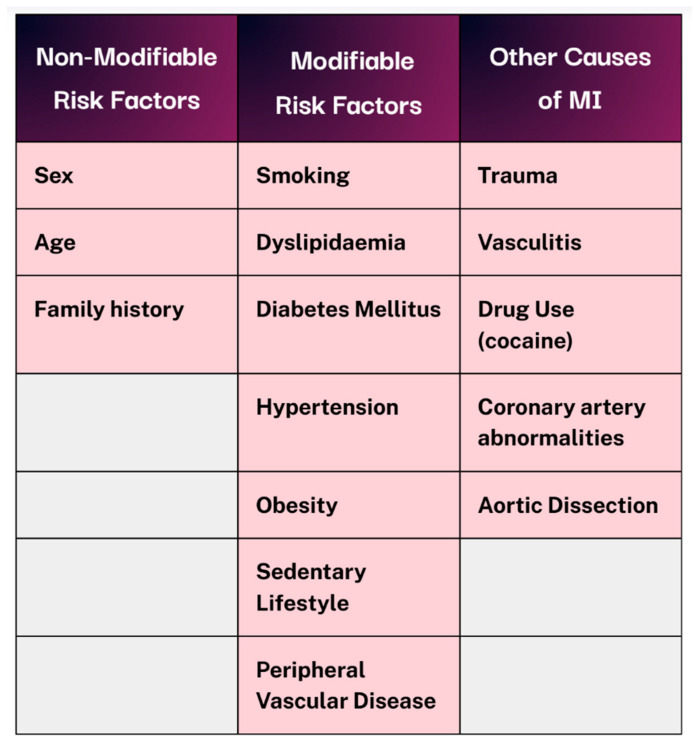
Non-modifiable vs modifiable risk factors and alternative causes of MI. Created in Canva. Adapted from [1].

Acute coronary syndrome (ACS) encompasses a range of conditions, including ST-elevation myocardial infarction (STEMI), non-ST elevation myocardial infarction (NSTEMI), and unstable angina. The latter two are distinguished primarily through cardiac biomarker analysis. As a symptomatic manifestation of coronary heart disease (CHD), ACS contributes to an estimated 9 million deaths globally each year [2] and is responsible for 12% loss of disability-adjusted life-years worldwide [3]. Risk factors for acute MI can be categorised into non-modifiable and modifiable groups, as illustrated in Figure 1. Beyond the immediate infarction, MI can result in lasting myocardial damage and a cascade of complications due to impaired systolic and diastolic functions. Therefore, timely reperfusion and the rapid restoration of coronary blood flow remain critical in improving both short- and long-term outcomes [4].

## 2. Diagnosis

Patients may present with a range of acute symptoms, such as chest pain radiating to the jaw/left arm, tightness, burning, or a sensation of heaviness, raising suspicion for ACS [4,5]. A key priority is distinguishing cardiac from non-cardiac causes of chest pain through a thorough history and clinical examination to prevent misdiagnosis or delays in initiating appropriate treatment. Older adults, individuals with diabetes, and female patients may present with atypical or non-specific symptoms, necessitating a heightened index of suspicion [4].

On examination, patients may appear visibly distressed and diaphoretic, often exhibiting tachycardia or other arrhythmias suggestive of underlying electrical disturbances. In cases of aortic dissection, asymmetrical pulses or blood pressure differences among limbs, along with severe back pain, may be present. Fever and tachypnoea can indicate a systemic inflammatory response. Elevated jugular venous pressure and distended neck veins suggest right ventricular failure and raised central venous pressure. Additionally, lateral displacement of the apical impulse and a soft first heart sound (S1) on auscultation may be noted. Pulmonary findings such as wheezing or crackles point to pulmonary oedema or congestive heart failure, often accompanied by bilateral peripheral oedema. Abdominal tenderness may suggest alternative diagnoses, including pancreatitis or gastritis [1,4,5].

The resting 12-lead ECG serves as the first-line diagnostic investigation in the evaluation of patients with ACS, enabling differentiation between STEMI from NSTEMI and unstable angina. Current guidelines recommend that an ECG be performed immediately and interpreted within 10 min of the patient’s arrival [4,6,7]. If STEMI is confirmed by persistent ST-segment elevation, alongside a raised troponin, prompt activation of the catheterisation laboratory for primary percutaneous coronary intervention (PCI) is essential. If STEMI is suspected, patients should be promptly triaged for emergency reperfusion without waiting for biomarkers [8]. In these cases, the interval from first medical contact to device activation should ideally be under 90 min [9]. Moreover, patients with ACS complicated by cardiogenic shock or haemodynamic instability require immediate revascularisation of the culprit vessel via PCI or coronary artery bypass graft (CABG), regardless of symptom onset timing [10].

**Figure 2:**
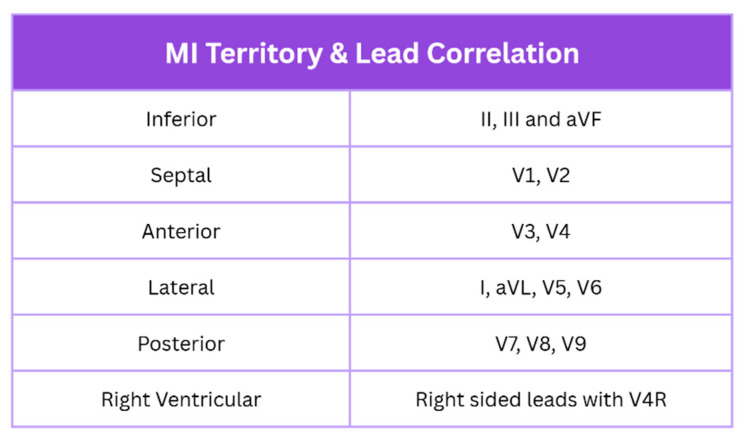
MI localisation with lead correlation on ECG. Created in Canva. Adapted from [1].

New ST elevation in two contiguous leads suggests ongoing coronary artery occlusion. Lead grouping consists of anterior/septal (V1–V4), inferior (II, III, and aVF), lateral (I, aVL, and V5–V6), posterior (V7–V9), and right ventricular, as shown in Figure 2 [4,11]. Criteria include ≥2.5 mm in men < 40, ≥2 mm in men > 40, and ≥1.5 mm in women regardless of age. This may be accompanied by ≥1 mm ST elevation in other leads, provided there is no left ventricular hypertrophy or left bundle branch block. STEMI is typically marked by convex ST elevation with reciprocal ST depression, helping distinguish it from pericarditis. STEMI equivalents include posterior MI (ST depression with tall R waves in V1–V3) and left main occlusion (widespread ST depression with ST elevation in aVR).

Cardiac troponin, preferably high-sensitivity (hs-cTn), is the most specific biomarker for MI [12,13]. If initial troponin levels are inconclusive, hs-cTn should be remeasured within 1–2 h or at 3–6 h for conventional cardiac troponin assays [14]. These play a critical role in distinguishing NSTEMI from unstable angina, the latter lacking evidence of myocardial necrosis. The troponin I and T isoforms typically peak around 12 h after onset and remain elevated for up to 7 days. If troponin levels remain below the 99th percentile, the diagnosis leans towards unstable angina, in contrast to the characteristic rise and fall observed in NSTEMI [15,16]. ECG findings in NSTEMI can vary widely, ranging from transient ST-segment elevation or depression to hyperacute or biphasic T waves, T wave inversions, or even a completely normal ECG, highlighting the importance of serial testing and clinical correlation [17]. Acute myocardial injury is defined by a rise and/or fall in cTn above the 99th percentile URL, accompanied by clinical evidence of acute myocardial ischaemia.

According to the Fourth Universal Definition of Myocardial Infarction, MI can be classified into five types. Type 1 is spontaneous MI due to atherosclerotic plaque rupture, while Type 2 results from an oxygen supply–demand mismatch without acute coronary thrombosis, prevalent in critically ill and elderly patients. Type 3 refers to sudden cardiac death with presumed MI before biomarker analysis/confirmation. Type 4 encompasses PCI-related infarctions, as follows: Type 4a (troponin rise >5× the 99th-percentile URL with new evidence of myocardial ischaemia), Type 4b (stent thrombosis), and Type 4c (restenosis). Finally, Type 5 is associated with coronary artery bypass grafting [18]. In NSTEMI patients undergoing PCI, recognising Type 4a and Type 5 periprocedural infarctions has been highlighted as key to aid risk stratification, carrying significant prognostic implications [19].

## 3. Initial Treatment

ECG-guided risk stratification allows for timely triage and optimises treatment initiation. Patients diagnosed with STEMI should be urgently transferred to the nearest PCI centre. In the absence of ST elevation, those with ongoing ischaemic symptoms consistent with NSTEMI should be managed according to the NSTEMI pathway to prevent complications.

**Figure 3:**
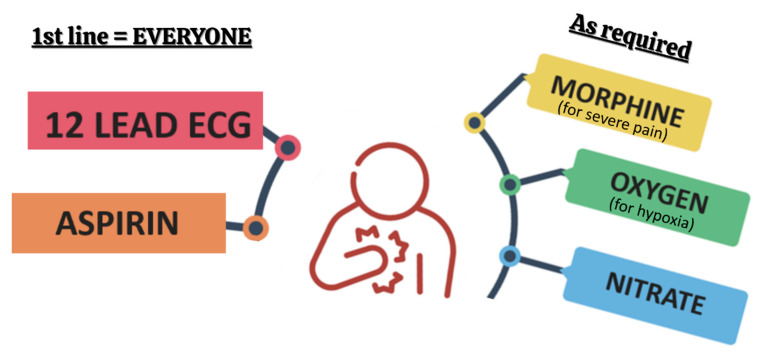
Initial management of patients with suspected MI. Created in Canva.

In the acute setting, the priority for both STEMI and NSTEMI patients is the prompt administration of a 150–300 mg oral-loading dose of aspirin (non-enteric coated). If oral administration is not feasible, 75–250 mg can be given intravenously [4]. Patient comfort should also be addressed, including oxygen therapy if saturations fall below 90% [20], along with anti-emetics, nitrates, and appropriate analgesia, as shown in Figure 3 [4,6].

Hofmann et al. demonstrated that routine oxygen supplementation in non-hypoxaemic patients with suspected MI (SpO_2_ ≥ 90%) offers no clinical benefit and is, therefore, not recommended [21]. However, studies in canine models have shown a significant reduction in infarct size and improved left ventricular ejection fraction with oxygen therapy followed by reperfusion [22]. These findings highlight the need for further research to assess the potential benefits of increased inspired oxygen concentrations in patients undergoing reperfusion therapy.

Intravenous access should be established promptly to enable effective pain management for severe chest pain, often requiring potent opioids. Diamorphine (2.5–5 mg) or morphine (5–10 mg) are commonly used due to their combined analgesic and anxiolytic effects [4]. A multicentre randomised trial, by Charpentier et al., involving 684 patients found that nitrous oxide/oxygen with IV acetaminophen was less effective than morphine in controlling chest pain in patients with STEMI [23]. However, opioids are associated with side effects such as nausea and vomiting, necessitating concurrent anti-emetics like cyclizine. Morphine has also been shown to delay activity of oral antiplatelet in STEMI patients by slowing gastrointestinal absorption [24] and may increase the frequency of arterial hypoxaemia in the first 24 h post-MI [25].

Sublingual nitrates alleviate ischaemic symptoms by reducing myocardial workload and oxygen demand through decreased preload and afterload [26]. Though used for over a century, nitrates are contraindicated in certain situations, including hypotension, marked bradycardia or tachycardia, right ventricular infarction, severe aortic stenosis, and recent use of phosphodiesterase-5 inhibitors within the past 24–48 h [5,6].

Patients with ACS and acute or chronic anaemia should also be considered for blood transfusions, aiming for haemoglobin level of ≥10 g/dL [27].

## 4. Acute Management of STEMI

All patients with suspected STEMI should be offered a 300 mg loading dose of aspirin [4], unless contraindicated—such as in cases of recent gastrointestinal bleeding, a history of severe hypersensitivity, or prior intracranial haemorrhage [26]. Reperfusion eligibility should be assessed universally, without bias to age, sex, ethnicity, or level of consciousness.

For patients who are not candidates for immediate reperfusion therapy, medical management centres around dual antiplatelet therapy, typically ticagrelor in combination with aspirin. In individuals with a heightened bleeding risk, clopidogrel with aspirin or aspirin monotherapy may be considered. A cardiology review should follow, including evaluation of left ventricular function.

If the patient presents within 12 h of symptom onset and PCI is achievable within 120 min, primary PCI should be performed. PCI may still be appropriate beyond 12 h in the presence of ongoing ischaemia or cardiogenic shock. Prior to PCI, prasugrel in conjunction with aspirin is given, unless the patient is taking oral anticoagulants, in which case clopidogrel should be used instead [4]. Radial access is generally favoured over femoral [28,29], with administration of unfractionated heparin with bailout glycoprotein inhibitors or bivalirudin [30]. Drug-eluting stents are preferred in cases requiring stenting [31]. For patients with multivessel disease in the absence of cardiogenic shock, the goal is complete revascularisation [32]. Initial PCI should be of the culprit vessel, followed by PCI or CABG of significantly stenosed non-infarct-related vessels [33]. The GUSTO-IIb trial demonstrated a significantly greater restoration of normal coronary flow with primary PCI compared to fibrinolysis [34].

When PCI is not feasible within 120 min, fibrinolytic therapy should be promptly administered alongside an appropriate antithrombin. Robust randomised trials have shown that fibrinolysis can prevent approximately 30 early deaths per 1000 patients if given within 6 h of symptom onset [35]. As with PCI, prasugrel and aspirin remain the first-line antiplatelet regimen with fibrinolysis unless contraindicated due to bleeding risk [4].

**Figure 4:**
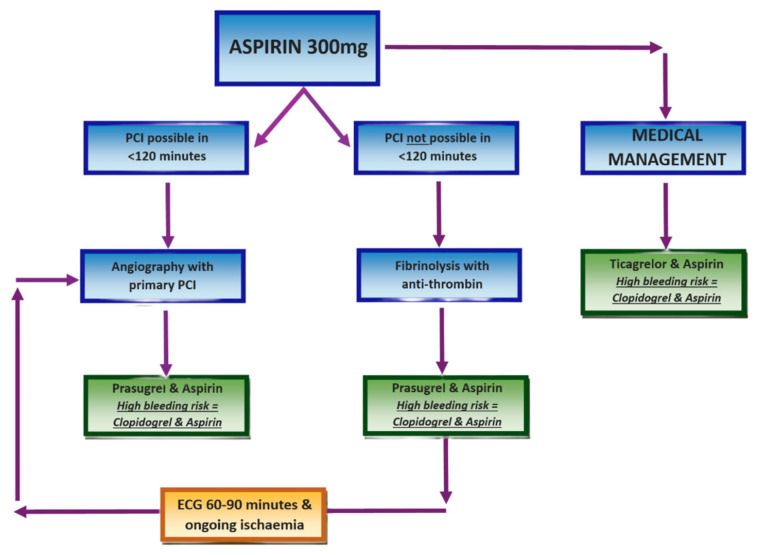
STEMI management protocol. Created in Canva. Adapted from [32].

An ECG should be repeated 60–90 min post-fibrinolysis to assess therapeutic response. Even if there is ≥50% resolution in ST-segment elevation along with relief of chest pain, angiography should be performed within 2–24 h [36]. However, in cases of failed lysis, re-occlusion, or re-infarction, indicated by persistent or recurrent ST elevation, repeat fibrinolysis is discouraged. Instead, immediate angiography with follow-on PCI is indicated [37], as outlined in Figure 4.

Irrespective of the initial strategy, all STEMI patients must be enrolled in a structured cardiac rehabilitation programme and receive comprehensive secondary prevention to optimise long-term outcomes [32,38].

## 5. Acute Management of NSTEMI and Unstable Angina

As in STEMI, patients presenting with NSTEMI should be administered a 300 mg loading dose of aspirin, to be continued indefinitely unless contraindicated [4]. Fondaparinux is the preferred initial antithrombin agent, unless there is a high bleeding risk or the patient requires immediate angiography [39]. In patients with impaired renal function (creatinine > 256 µmol/L), unfractionated heparin should be considered, with dosage adjusted according to coagulation parameters [40].

Risk stratification should be performed using the Global Registry of Acute Coronary Events (GRACE) score, incorporating clinical history, physical examination findings, ECG results, and key biochemical markers (troponin, creatinine, glucose, and haemoglobin). For patients with a predicted 6-month mortality of ≤3% (low risk), conservative management without angiography is appropriate. These patients should receive dual antiplatelet therapy with ticagrelor and aspirin, or clopidogrel and aspirin if there is an elevated bleeding risk. If clinical signs of ischaemia persist, angiography with follow-on PCI should be considered [4,32,33].

**Figure 5:**
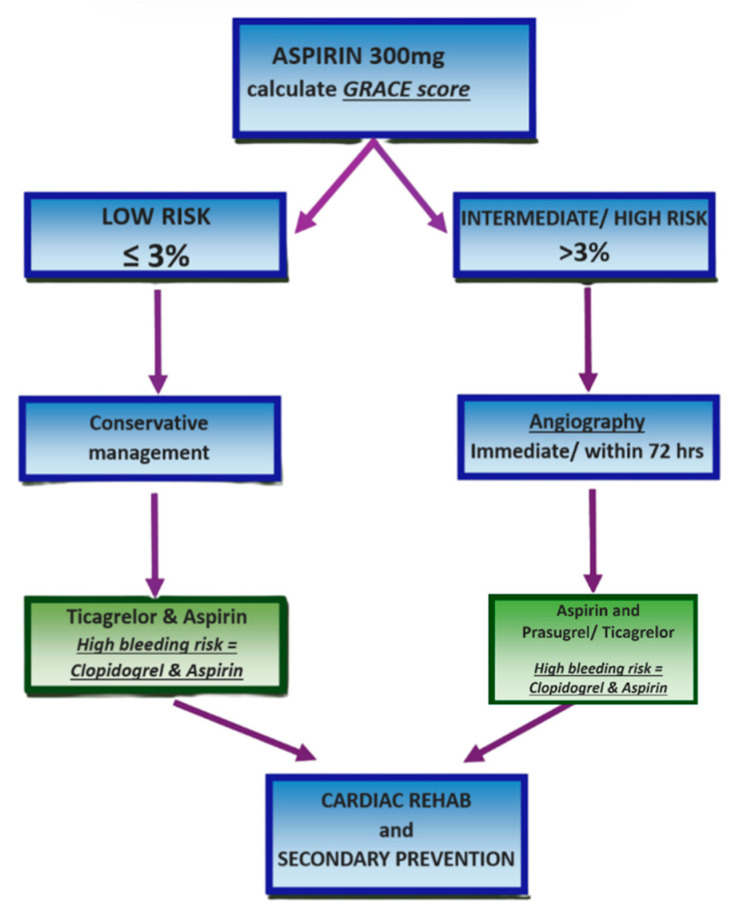
NSTEMI and unstable angina management protocol. Created in Canva. Adapted from [32].

Conversely, patients with a GRACE score > 3% (intermediate to high risk) should undergo urgent angiography if they exhibit refractory angina or haemodynamic or electrical instability [33]. If stable, angiography with or without PCI should be performed within 72 h, provided there are no contraindications such as active bleeding. These patients may be treated with prasugrel or ticagrelor alongside aspirin, with clopidogrel preferred for those on concurrent oral anticoagulation. The PCI protocol should follow established STEMI guidelines, including the use of unfractionated heparin and drug-eluting stents where indicated. This strategy of delayed PCI has been associated with improved in-hospital mortality and reduced length of stay in NSTEMI patients [1]. Finally, all patients should undergo left ventricular function assessment and be referred for comprehensive cardiac rehabilitation and secondary prevention, as illustrated in Figure 5 [32,41].

## 6. Secondary Prevention

Following myocardial infarction, patients should be commenced on dual antiplatelet therapy (DAPT) for 12 months, after which lifelong aspirin at 75 mg is recommended, unless contraindicated. Ticagrelor or prasugrel with aspirin are offered as DAPT; however, clopidogrel should be used in individuals with active bleeding or a history of intracranial haemorrhage. In patients at elevated bleeding risk, DAPT may be shortened to 6 months, whereas those at high risk of recurrent cardiovascular events may benefit from extended therapy beyond 12 months [4,5,6]. In-hospital bleeding reflects patient frailty in patients with ACS and should prompt careful monitoring during follow-up [42]. For individuals at risk of gastrointestinal bleeding, a proton pump inhibitor (PPI) should be co-prescribed. As clopidogrel interacts with omeprazole, lansoprazole is the preferred alternative [43].

Beta-blockers should be initiated within 24 h, provided there are no contraindications such as heart failure, hypotension, atrioventricular block, or reactive airway disease [44]. Additionally, ACE inhibitors are recommended for those with left ventricular ejection fraction (LVEF) < 40%, hypertension, diabetes, or chronic kidney disease. The EPHESUS trial demonstrated significant benefits of mineralocorticoid receptor antagonists in patients with heart failure or diabetes [45].

A meta-analysis showed that sodium–glucose co-transporter 2 inhibitors (SGLT2is) significantly reduce major adverse cardiovascular events (MACEs) in diabetic patients [46]. Post-MI, SGLT2i like empagliflozin have improved outcomes and lowered BNP levels in heart failure patients, as evidenced by the EMMY and DAPA-MI trials [47,48]. Recent evidence suggests that SGLT2i may also reduce infarct size [49] and help prevent atrial fibrillation [50], especially in patients without pre-existing heart failure.

High-intensity statins should be initiated promptly post-MI, irrespective of baseline lipid levels, to optimise long-term cardiovascular outcomes [43,51]. If patients are on maximal tolerated statin therapy, adding a non-statin medication (PCSK9 inhibitors or inclisiran) can further reduce risk of MACEs [52]. Additionally, glucagon like peptide 1 receptor agonists have been shown in meta-analyses to lower MACE and cardiovascular mortality [53]. The COLCOT and LoDoCo2 trials demonstrated that anti-inflammatory agents such as colchicine reduce MACE and cardiovascular deaths. However, there is a noted increase in non-cardiovascular mortality [54,55].

Beyond pharmacological management, comprehensive lifestyle interventions are essential. Smoking cessation counselling should be routinely offered, with evidence indicating a 50% reduction in recurrent myocardial infarction risk [56]. Dietary modifications, particularly adherence to a Mediterranean style diet rich in fruits, vegetables, and oily fish, are advised to reduce total fat intake and encourage the substitution of saturated with polyunsaturated fats [57]. Structured cardiac rehabilitation, including stress reduction strategies and regular cardiopulmonary exercise, should commence within 10 days post-discharge [58,59]. Additionally, meticulous control of contributory co-morbidities, such as hypertension and diabetes, is imperative to mitigate future cardiovascular events [60].

## 7. Post-MI Complications

**Figure 6:**
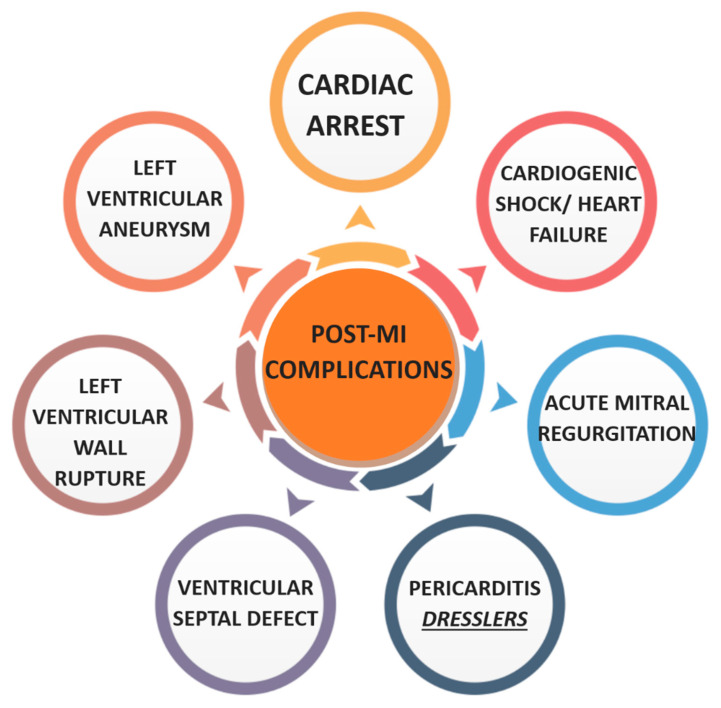
Diagram of post-MI complications. Created in Canva.

A multitude of complications may ensue following MI, as illustrated in Figure 6. Sudden cardiac death remains a significant cause of mortality. This risk is attributed to a constellation of pathophysiological mechanisms including neurohormonal dysregulation, myocardial scar formation with impaired remodelling, autonomic imbalance, and evolving heart failure [61]. Patients with ACS and reduced LVEF < 40% have increased risk of life-threatening arrhythmias [62]. Implantable cardioverter-defibrillators post-MI are therefore recommended for this cohort [63].

Cardiac arrest is predominantly due to malignant arrhythmias such as ventricular fibrillation and can occur within the first 30 days post-MI. Resuscitated cardiac arrest patients with evidence of STEMI should be considered for transfer to a PCI centre [64]. In contrast, immediate angiography is not recommended in post-arrest NSTEMI patients, as no clinical benefit has been demonstrated [65].

Cardiogenic shock represents another critical complication, with 30-day mortality rates reaching approximately 30% and escalating to 50% within one year. The CULPRIT-SHOCK trial advocates for immediate coronary angiography and revascularisation of the infarct related artery in patients with MI associated cardiogenic shock, demonstrating an 8% reduction in 30-day mortality [66]. Myocyte necrosis and the resultant loss of viable myocardium contribute to diminished ejection fraction and ventricular dysfunction. In refractory cardiogenic shock, mechanical circulatory support such as Impella or ECMO may be indicated to maintain cardiac output and end-organ perfusion while definitive therapy is instituted [67].

Chronic heart failure may ensue due to sustained myocardial impairment. While loop diuretics can be utilised for symptomatic relief in cases of fluid overload, they offer no survival benefit. Long-term prognosis is significantly improved by the early introduction of ACE inhibitors and beta-blockers [68].

Structural complications post-MI include the formation of left ventricular aneurysms and pseudoaneurysms, secondary to ischaemic damage and myocardial wall weakening. Pseudoaneurysms, which arise from contained myocardial rupture within pericardial adhesions, often manifest with signs of congestive heart failure, chest discomfort, and dyspnoea. These may be accompanied by sudden cardiac death. Electrocardiography may reveal persistent ST-segment elevation, while radiography often demonstrates cardiomegaly. Due to their association with thrombus formation and consequent stroke risk, anticoagulation is essential alongside consideration for percutaneous repair [69].

Acute mitral regurgitation secondary to papillary muscle rupture, though less common in the modern era, remains associated with high mortality. It typically presents with acute pulmonary oedema progressing to cardiogenic shock. An early to mid-systolic murmur may be auscultated. Risk factors include advanced age, female sex, prior heart failure, and chronic kidney disease. Initial management may require vasoactive agents and mechanical ventilation, with urgent surgical assessment to determine the need for mitral valve replacement or concurrent coronary artery bypass grafting [70,71].

Left ventricular free wall rupture is considered the most common mechanical complication post-MI. Its precise incidence remains uncertain, largely due to its association with out of hospital sudden cardiac death, typically occurring within 1–2 weeks of the infarction [72]. Delayed reperfusion has been identified as a significant risk factor [73]. Clinical features often include pulsus paradoxus, raised jugular venous pressure, muffled heart sounds, and signs of tamponade physiology. Emergency management entails immediate pericardiocentesis followed by definitive surgical repair [74].

Ventricular septal defects (VSDs) are another post-infarction mechanical complication. Usually arising within the first week, they can present variably from a pansystolic murmur to frank cardiogenic shock due to left to right shunting. Clinical signs include dyspnoea, hypotension, and cool extremities. Transthoracic echocardiography remains the diagnostic modality of choice to assess the size and direction of shunt. Without prompt surgical intervention, VSDs are associated with exceptionally high mortality [72,75].

Pericarditis post-MI may be classified as early (within 48 h) or late, with the latter often referred to as Dressler’s syndrome [76,77]. Early pericarditis typically presents with pleuritic chest pain exacerbated by lying supine, a pericardial friction rub on auscultation, and pericardial effusion on echocardiography. Dressler’s syndrome, occurring 2–6 weeks post-MI, is an immune-mediated phenomenon characterised by fever, elevated ESR, and pericardial effusion [78]. Management involves NSAIDs with a PPI and colchicine to prevent recurrence. For patients already on antiplatelets, high-dose aspirin may be used for 2–4 weeks with gradual tapering. In cases where NSAIDs or aspirin are contraindicated, glucocorticoids can be administered [79].

## 8. Discussion

Over the past century, the field of interventional cardiology has undergone transformative advancements, with procedures such as PCI, transcatheter aortic valve implantation, and left atrial appendage closure markedly improving patient outcomes in the management of cardiovascular disease [80]. In parallel, the introduction and widespread adoption of potent antithrombotic agents and statins have significantly contributed to reducing low-density lipoprotein levels, thereby lowering the incidence of major vascular events, including myocardial infarction [81]. The emergence of the cholesterol efflux and inflammatory hypothesis has further broadened the therapeutic landscape, providing new molecular targets aimed at modulating atherosclerotic progression and enhancing patient prognosis [82]. Nonetheless, persistent challenges remain. A study by Jernberg et al. demonstrated that despite declining mortality rates, the risk of recurrent ischaemic cardiovascular events within the first year following myocardial infarction remains substantially high [83].

Further research is warranted to innovate novel technologies that facilitate earlier detection of acute cardiac events. The integration of artificial intelligence holds considerable promise in augmenting existing diagnostic modalities and enhancing prognostic accuracy. AI-driven tools could be used to enhance ECG interpretation, detecting subtle ST-segment changes and early patterns of ischaemia. Incorporating AI into routine practice has the potential to increase diagnostic accuracy and risk stratification, accelerate decision making, and tailor management strategies for patients with ACS [84].

In conclusion, the management of MI necessitates a prompt, comprehensive, and multidisciplinary approach. Timely and accurate diagnosis, primarily through ECG and troponin evaluation, enables differentiation between STEMI and NSTEMI to avoid delay in reperfusion. Robust secondary prevention remains paramount, encompassing lifelong antiplatelet therapy, structured lifestyle intervention, and rigorous optimisation of cardiovascular risk factors and comorbid conditions. Moreover, heightened clinical vigilance for post-infarction complications is essential, given their profound impact on long-term prognosis despite the substantial strides made in contemporary cardiovascular care. A patient-centred approach with shared decision making is essential to aligning with patient expectations and enhancing outcomes and overall experience.
